# Patterns of eHealth Website User Engagement Based on Cross-site Clickstream Data: Correlational Study

**DOI:** 10.2196/29299

**Published:** 2021-08-13

**Authors:** Jia Li, Kanghui Yu, Xinyu Bao, Xuan Liu, Junping Yao

**Affiliations:** 1 School of Business East China University of Science and Technology Shanghai China; 2 Xi'an Research Institute of High Technology Xi'an China

**Keywords:** engagement, clickstream data, cross-site visit, platform, channel, mobile phone

## Abstract

**Background:**

User engagement is a key performance variable for eHealth websites. However, most existing studies on user engagement either focus on a single website or depend on survey data. To date, we still lack an overview of user engagement on multiple eHealth websites derived from objective data. Therefore, it is relevant to provide a holistic view of user engagement on multiple eHealth websites based on cross-site clickstream data.

**Objective:**

This study aims to describe the patterns of user engagement on eHealth websites and investigate how platforms, channels, sex, and income influence user engagement on eHealth websites.

**Methods:**

The data used in this study were the clickstream data of 1095 mobile users, which were obtained from a large telecom company in Shanghai, China. The observation period covered 8 months (January 2017 to August 2017). Descriptive statistics, two-tailed *t* tests, and an analysis of variance were used for data analysis.

**Results:**

The medical category accounted for most of the market share of eHealth website visits (134,009/184,826, 72.51%), followed by the lifestyle category (46,870/184,826, 25.36%). The e-pharmacy category had the smallest market share, accounting for only 2.14% (3947/184,826) of the total visits. eHealth websites were characterized by very low visit penetration and relatively high user penetration. The distribution of engagement intensity followed a power law distribution. Visits to eHealth websites were highly concentrated. User engagement was generally high on weekdays but low on weekends. Furthermore, user engagement gradually increased from morning to noon. After noon, user engagement declined until it reached its lowest level at midnight. Lifestyle websites, followed by medical websites, had the highest customer loyalty. e-Pharmacy websites had the lowest customer loyalty. Popular eHealth websites, such as medical websites, can effectively provide referral traffic for lifestyle and e-pharmacy websites. However, the opposite is also true. Android users were more engaged in eHealth websites than iOS users. The engagement volume of app users was 4.85 times that of browser users, and the engagement intensity of app users was 4.22 times that of browser users. Male users had a higher engagement intensity than female users. Income negatively moderated the influence that platforms (Android vs iOS) had on user engagement. Low-income Android users were the most engaged in eHealth websites. Conversely, low-income iOS users were the least engaged in eHealth websites.

**Conclusions:**

Clickstream data provide a new way to derive an overview of user engagement patterns on eHealth websites and investigate the influence that various factors (eg, platform, channel, sex, and income) have on engagement behavior. Compared with self-reported data from a questionnaire, cross-site clickstream data are more objective, accurate, and appropriate for pattern discovery. Many user engagement patterns and findings regarding the influential factors revealed by cross-site clickstream data have not been previously reported.

## Introduction

### Background

Providing and delivering web-based services is a major trend in the digital transformation of health services [1]. Currently, increasingly more users obtain health information, consult a physician, purchase drugs, or self-manage wellness from smartphones. As a result, understanding user behavior on smartphones is becoming increasingly important. Owing to differences in screen size and mobility, users behave differently on smartphones than on desktop computers [2]. Therefore, it is relevant to investigate eHealth website usage behaviors such as user engagement from smartphones.

User engagement is a key variable for eHealth websites [3]. The sizable demand for web-based health services has led to a large number of eHealth websites, which makes competition extremely fierce. According to the IQVIA Institute, more than 318,000 health apps are now available on top app stores worldwide, with more than 200 health apps being added each day [4]. As a result, it is increasingly difficult to obtain sufficient engagement for users on specific health websites. In addition, health care is a relatively low-frequency need compared with social networking, news reading, or web-based shopping. Users visit health websites or apps only when they have health concerns. All these factors make achieving sufficient user engagement on eHealth websites a difficult task.

Although many previous studies have investigated engagement patterns [5-9] and engagement interventions [3,10-15] on eHealth websites, most of them only focus on a single website. As a result, the research findings can only be applied to the corresponding categories of the eHealth website. However, the patterns of user engagement (eg, market share, penetration, intensity, variety, time trends, loyalty, and cross-site visits) on all eHealth websites and the factors that influence user engagement on all eHealth websites need to be examined. In addition, the links among the different categories of eHealth websites are largely unknown. For example, questions such as how users visit multiple eHealth websites simultaneously or how one type of eHealth website can provide referral traffic for other types of eHealth websites have not been answered by previous studies. By solving these questions, we can better understand user engagement behavior from a holistic view and keep users more engaged in different types of eHealth websites.

### Literature Review

User engagement on eHealth websites has received considerable attention in recent years. A review of the literature suggests two main research streams investigating engagement patterns and engagement interventions. The first research stream is descriptive in nature. The areas investigated include diabetes management [5,6], mental health management [7], pain management [8], and health information dissemination [9]. For diabetes management, Böhm et al [5] found that although more women used the app, they engaged significantly less with it. Older people and users who were recently diagnosed tended to use apps more actively. Glasgow et al [6] investigated engagement patterns on diabetes self-management websites. They found that participants visited the website fairly often and used all of the theoretically important sections, but engagement decreased over 4 months. For mental health management, Baumel et al [7] found that daily minutes of use were significantly higher for mindfulness, meditation and peer support apps than for apps incorporating other techniques (tracker, breathing exercise, and psychoeducation). The median 15-day and 30-day retention rates of the app were 3.9% and 3.3%, respectively, indicating that only a small portion of users actually used the apps for a long period. For pain management, Rahman et al [8] found that although most users of the app reported being female, male users were more likely to be highly engaged in the app. Users in the most engaged clusters self-reported a higher number of pain conditions, a higher number of current medications, and a higher incidence of opioid usage. For health information dissemination, Zhang et al [9] investigated the user engagement of health information disseminated by Chinese provincial centers for disease control and prevention on WeChat. They found that the median number of reads was 551.5 and the median number of likes was 10. Article content, article type, communication skills, number of marketing elements, and article length were associated with the reading and liking levels. However, title type was only associated with liking level.

The second research stream focuses on designing interventions to improve user engagement. System design [3,10], social support [11,12], gamification [13,14], and channels [15] are the most frequently investigated interventions to promote user engagement. For the system design, Baumel and Kane [10] found that therapeutic persuasiveness, therapeutic alliance, visual design, and content predict an increase in user engagement with eHealth interventions. Wei et al [3] investigated which design features improved user engagement with mobile health interventions. They identified the following seven themes that influenced user engagement: personalization, reinforcement, communication, navigation, credibility, message presentation, and interface esthetics. For social support, Kashian and Jacobson [11] found that optimal social support and tie strength were positively related to engagement. In addition, the more engaged members were, the more positive their health expectations were. Wang et al [12] revealed that the amount and match of received support were positive and significant predictors of new users’ continued engagement. For gamification, Edney et al [13] found that the inclusion of gamified features enhanced engagement in an app-based physical activity intervention. Comello et al [14] found that a game-inspired infographic showed the potential to outperform a traditional format for comprehension and decreased cognitive load while not underperforming on engagement (eg, attitudes and emotional tone). With regard to the channel used, Brusk and Bensley [15] compared the impact of mobile versus fixed devices on user engagement key performance indicators. They found that eight user characteristics (lessons completed, race, ethnicity, language, state of residence, pregnancy status, beginning stage of change, and preferred nutrition education method) were significantly related to various key performance indicator differences between mobile and fixed device access. Surprisingly, their results suggest that nonmobile users are more likely to engage.

The review results listed above indicate that extant studies on user engagement in eHealth only focus on a single website. A higher level of analysis that provides a complete picture of how users engage in different types of eHealth websites is still lacking. Although the meta-analysis allows multiple websites to be considered together, existing review studies on this topic still focus on a single category [3]. The link between the different categories of eHealth websites is missing. To bridge this gap, we investigate user engagement behavior on all eHealth websites based on cross-site clickstream data.

### Research Questions

To bridge this research gap, we provide an analysis of user engagement on all eHealth sites with cross-site clickstream data in this study. Following the two research streams on user engagement [5-15], we focus on both user engagement patterns and engagement interventions.

First, we are interested in investigating user engagement patterns on all eHealth websites. More specifically, we will provide a framework for understanding the engagement patterns on eHealth websites. The framework includes the taxonomy of eHealth websites, market share, penetration, engagement intensity, engagement variety, day and hour trends, customer loyalty, and cross-site engagement. The taxonomy of eHealth websites is necessary because there are too many individual eHealth websites that cannot be covered in a single study. In addition, working on specific websites makes it difficult to reach a conclusion with general significance. Market share and penetration are included because they can jointly describe the market status quo and potential for that type of eHealth website (eg, a small market share with a high penetration usually means a great potential). Intensity, variety, time trend, loyalty, and cross-site behavior are included because they describe different aspects of user engagement. Therefore, the first research question (RQ) is as follows: What are the overall patterns of user engagement on multiple eHealth websites on smartphones (RQ1)?

Second, we are interested in identifying the factors that may influence user engagement on all eHealth websites. Following the Person, Environment, and Technology framework [16], user behavior in information systems can be well explained by personal, environmental, and technological factors. For personal factors, we focused on sex because male and female users exhibit sizable differences in their web behavior [17]. For environmental factors, we focused on income because the literature has suggested that behavior differences exist between high-income and low-income users [17]. For technological factors, we focused on the platform (operating system of the mobile phone) and channel (mobile browsers or mobile apps). Platform was included because there are many differences between iOS and Android communities, such as the number of eHealth apps and the percentage of free apps, which may lead to different engagement behaviors on eHealth websites [18]. Channel was also included because an app channel provides better experience than a browser channel, and such an advantage may lead to more intensive user engagement. To sum up, we investigate how the platform, channel, sex, and income influence user engagement on eHealth websites. Therefore, the second RQ is as follows: How do factors such as the platform, channel, sex, and income influence user engagement on multiple eHealth websites on smartphones (RQ2)?

This study has several practical implications. First, our clickstream data analysis indicates that the visit penetration for eHealth websites is very low, and users usually concentrate only on one or two websites. However, eHealth websites are also characterized by relatively high user penetration. Therefore, eHealth websites should have great market potential. One possible way to increase user engagement on more eHealth websites is to provide cross-site recommendations. Medical websites are ideal sources for effectively providing referral traffic for lifestyle and e-pharmacy websites. However, managers must be cautious that the opposite may not be true. Understanding the asymmetric nature of cross-site browsing can help managers improve the effects of cross-site recommendations.

Second, the findings of this research show that Android users are more engaged in eHealth websites than iOS users, partly because more health apps are available on the Android platform, and the Android platform has a higher percentage of free apps than the iOS platform. Therefore, managers of the iOS platform should encourage developers to develop more health apps (especially free apps or apps with in-app purchase features) in the future.

Third, the results of this study suggest that app users are, on average, 4.5 times more engaged than browser users. Therefore, all eHealth websites should provide apps for both Android and iOS platforms. The managers of eHealth websites should also encourage users to download their apps and urge users to access their websites from apps instead of browsers.

## Methods

The data used in this study are the access log data of 1095 4G users from a large telecom company in Shanghai, China. The observation period was 8 months (January 2017-August 2017). After removing confidential information (eg, telephone numbers), we obtained users’ internet access records on smartphones. Each access record contains the encrypted user ID, access time, mobile platform (mobile operating system), and URL visited. User demographic information such as encrypted user ID, sex, age, and monthly expenditures on mobile phones was also included in the data set.

The eHealth websites investigated in this study can be classified into the following three categories: medical, lifestyle, and e-pharmacy [19]. Medical websites provide medical information on specific diseases or treatments. Lifestyle websites provide health information on fitness, weight loss, health management, or beauty care. e-Pharmacy websites provide web-based pharmacy services. For each category, we included the top 79.99% (184,826/231,033) most visited websites as the targets in this study, as per the 80-20 rule (also known as the Pareto principle); 20% of websites accounted for 80% of visits. This allowed us to investigate a small number of websites and obtain good coverage of visits to all eHealth websites. By following this approach, the eHealth websites investigated in this study were identified; they are listed in Table 1. The indicators (ie, visits and visitors) in Table 1 were calculated based on all 373 users who visited the websites listed in Table 1 during the 8-month observation period (January 2017 to August 2017).

**Table 1:** The eHealth websites investigated in this study (in China; n=373).

Category and website		Domain name	Visits, n (%)	Visitors, n (%)
**Medical**				
	Good Doctor	haodf.com	44,202 (23.9)	56 (15.1)
	WeDoctor	guahao.com	37,734 (20.4)	38 (10.2)
	39 Health Net	39.net	23,007 (12.5)	42 (11.4)
	Ask Doctor Quickly	120ask.com	15,613 (8.5)	70 (18.9)
	Seeking Medical Advice	xywy.com	11,769 (6.4)	69 (18.6)
	Chunyu Doctor	chunyuyisheng.com	1684 (0.9)	9 (2.3)
**Lifestyle**				
	Mint Health	boohee.com	41,462 (22.4)	8 (2.1)
	Health Preserving	cndzys.com	4307 (2.3)	17 (4.6)
	So-Young	soyoung.com	1101 (0.6)	34 (9)
**e-Pharmacy**				
	Kang Aiduo Pharmacy	360kad.com	2639 (1.4)	11 (3)
	Jianke Pharmacy	jianke.com	1308 (0.7)	17 (4.7)

## Results

### User Engagement Patterns

The engagement patterns investigated in this study include market share, penetration, engagement intensity, engagement variety, day and hour trends, customer loyalty, and cross-site engagement.

#### Market Share

Market share is the percentage of the market that a single category controls based on the number of visits. The proportion of medical websites is relatively large and accounts for 72.51% (134,009/184,826) of the total, whereas the proportion of e-pharmacy websites is very small and accounts for only 2.14% (3947/184,826) of the total. The proportion is lifestyle websites is 25.36% (46,870/184,826).

This finding indicates that the greatest demand for eHealth websites is to obtain health knowledge and medical advice such as that on prevention, diagnosis, prognosis, and treatment plans. Lifestyle websites also received considerable market share, suggesting that the idea of health management is currently pervasive in China. However, the proportion of visits to e-pharmacy websites was relatively small. A possible reason for this is that the purchase of drugs is a low-frequency demand. Another possible reason is that e-pharmacies are not yet included in the scope of medical insurance in most areas of China. Lack of trust in e-pharmacy websites is also a reason.

#### eHealth Behavior Penetration

eHealth behavior penetration measures how user behaviors on eHealth websites compare with those of all web behaviors. We focus on two types of user behaviors (Table 2): visit penetration (the percentage of visits to eHealth websites with respect to the visits to all websites) and user penetration (the percentage of users who have ever visited an eHealth website). The results in Table 2 show that the visit penetration of eHealth behavior is quite low, suggesting that eHealth websites correspond to very low-frequency demand compared with all web-based demands (eg, social networking, reading news, and web-based shopping). Users will access eHealth websites only when they have health concerns.

The results in Table 2 also suggest that the user penetration of eHealth behavior is relatively high (142/373, 38.1%). Overall, 33.5% (124/373) of the users had visited a medical website, 12.7% (47/373) had visited a lifestyle website, and 6.1% (23/373) had visited an e-pharmacy website. Considering that we only include 11 top-ranked eHealth websites in this study, the actual user penetration rates will be higher than the estimation reported in Table 2. Therefore, eHealth is an application with low traffic but high user penetration, which is closely related to everyone and has great market potential.

**Table 2:** eHealth behavior penetration among three categories (n=373).

Category	Visit penetration, n (%)	User penetration, n (%)
Medical	134,009 (0.082)	124 (33.5)
Lifestyle	46,870 (0.029)	47 (12.7)
e-Pharmacy	3947 (0.002)	23 (6.1)

#### Engagement Intensity

**Figure 1:**
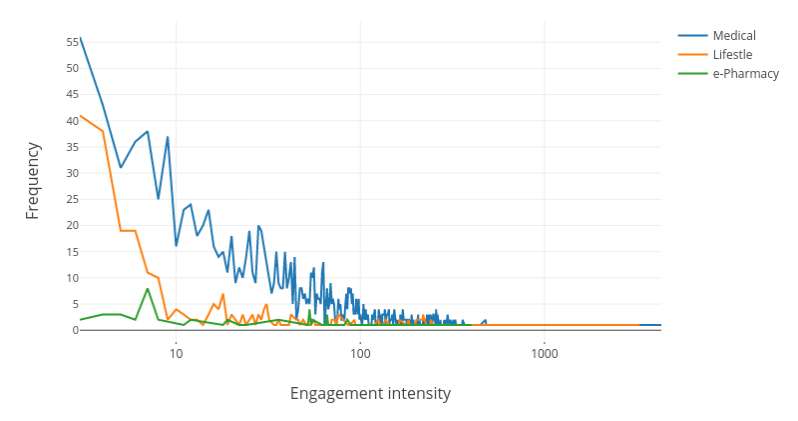
Visit intensity per day of the three categories of eHealth websites.

Engagement intensity is the number of visits to eHealth websites per session. In this study, we defined the length of a session as a day. Therefore, we measured engagement intensity as the number of visits to eHealth websites within a day. The engagement intensity patterns according to category are shown in Figure 1. As shown in Figure 1, the frequency decreased exponentially as the engagement intensity increased. The average engagement intensity is 105, indicating that a typical user interacts with these websites 105 times per day to fulfill their needs.

The results of the Kolmogorov-Smirnov test (D=0.025; *P*=.93) suggest that the distribution of engagement intensity follows a power law distribution [20]. That is, a large number of eHealth needs involve only a small number of visits, whereas a very small number of complex eHealth needs must be realized through a large number of visits. The mechanism of the power law distribution is the lack of natural growth constraints. The number of Facebook fans, the distribution of wealth in an unfair society, or the number of hits on web pages are all examples of data that follow a power law distribution. Visits to eHealth websites also lack constraints. Users can visit the website as many times as they want. However, a majority of health needs are simple. In most cases, users only need to visit eHealth websites 5-10 times to satisfy their needs.

#### Engagement Variety

Engagement variety measures the extent to which users visit different types of eHealth websites. In this study, engagement variety was measured by the number of distinct eHealth websites over 3 months (Table 3). The results in Table 3 suggest that most users (238/373, 63.8%) visited only 1 eHealth website within 3 months. On average, each user visits 1.5 of the eHealth websites in 3 months. Fewer than 40% (135/373, 36.2%) of users visit multiple eHealth websites. Among these users, 90% (121/135, 89.6%) visit only two to three websites, and very few users visit four or more websites.

**Table 3:** The distribution of engagement variety (n=373).

Number of websites accessed	Visitors, n (%)
1	238 (63.8)
2	76 (20.4)
3	45 (12.1)
4	7 (1.9)
5	5 (1.3)
6	2 (0.5)
7	0 (0)

This finding suggests that eHealth websites are highly isolated. Users have great inertia and pay attention to only one or two websites. For example, low engagement variety may be attributed to the fact that increasingly more eHealth websites provide one-stop services where users can meet almost all their health needs on one site. The low visit variety also suggests that the links among eHealth websites are insufficient. As a result, users from one website may not be aware of other websites for quite a long time.

#### Day of the Week Trends

**Figure 2:**
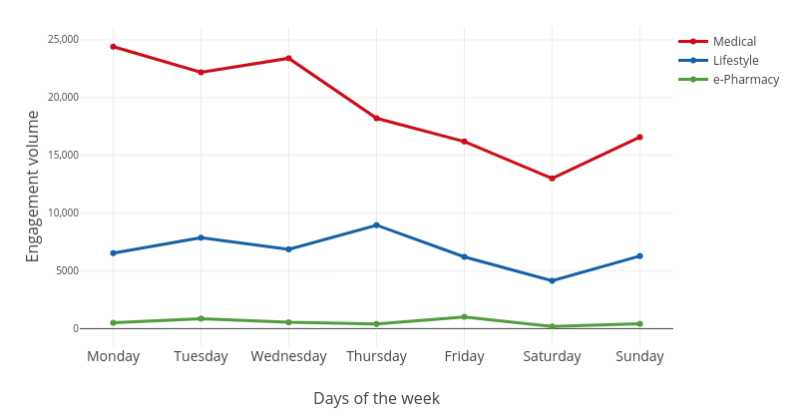
The fluctuation of the engagement volume in a week.

**Figure 3:**
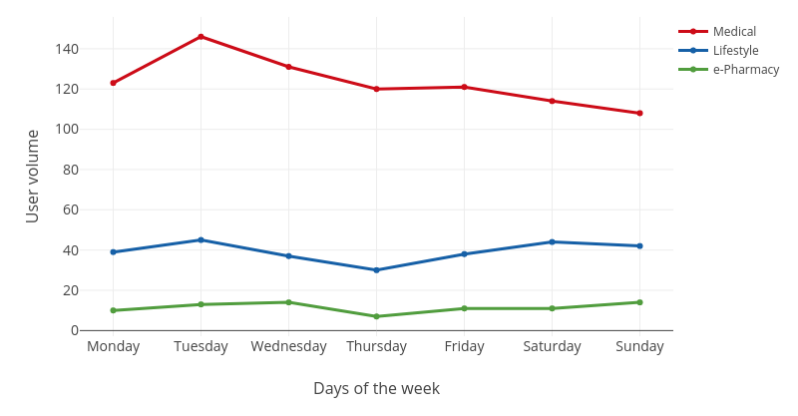
The fluctuation of the user volume in a week.

**Figure 4:**
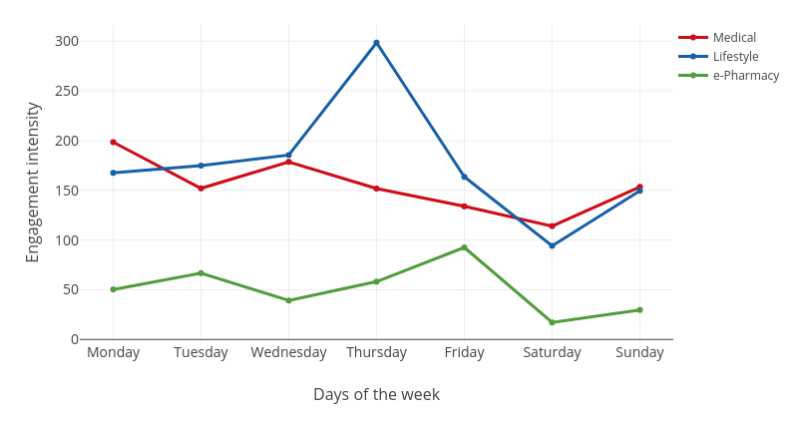
The fluctuation of the engagement intensity in a week.

We were interested in user engagement patterns at the week and day levels. For both the week and day levels, we observed the trends of the three key engagement variables (ie, engagement volume, user volume, and engagement intensity) over time. The engagement volume was measured by the number of visits. The user volume was measured by the number of unique users. The engagement intensity was measured by the number of visits per user. All the measures for engagement volume, user volume, and engagement intensity were based on 373 users who visited the websites listed in Table 1 (January 2017-August 2017). The engagement trends at the week level are shown in Figures 2-4.

For medical websites, there was more engagement from Monday to Wednesday, with the highest engagement volume and intensity seen on Monday. From Thursday to Saturday, the engagement volume and intensity decreased gradually until Sunday. The user volume was the highest on Tuesday, but the lowest on Sunday. In addition, the user volume of medical websites fluctuated more than that of the other two categories of websites.

For lifestyle websites, the engagement volume and intensity increased from Sunday to Thursday and then gradually decreased until Saturday. Engagement volume and intensity were the highest on Thursday and lowest on Saturday. However, the user volume on Thursday was the lowest in the week.

For e-pharmacy websites, the engagement volume, user volume, and engagement intensity were all the lowest compared with those of medical and lifestyle websites. The engagement volume and intensity were the highest on Friday but lowest on Saturday.

As shown in Figures 2-4, user engagement is generally high on weekdays but low on weekends. This finding is consistent with previous findings in the social network context that the posting of microblogs is usually more intensive on weekdays than on weekends [21]. One possible explanation is that users are more interested in offline relaxation activities on weekends.

#### Hour of the Day Trends

**Figure 5:**
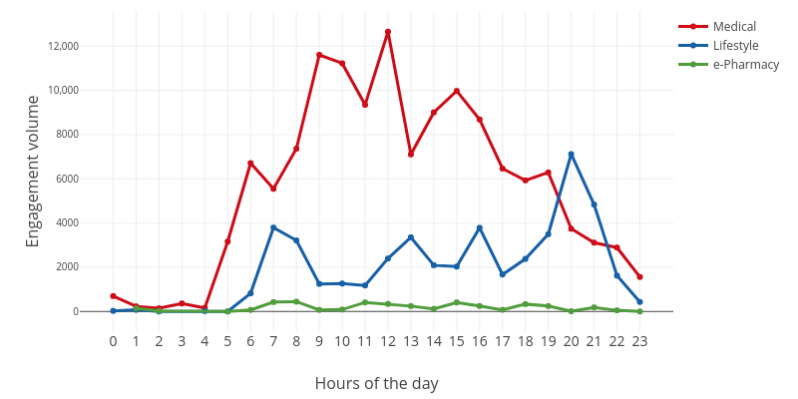
The fluctuation of the engagement volume in a day.

**Figure 6:**
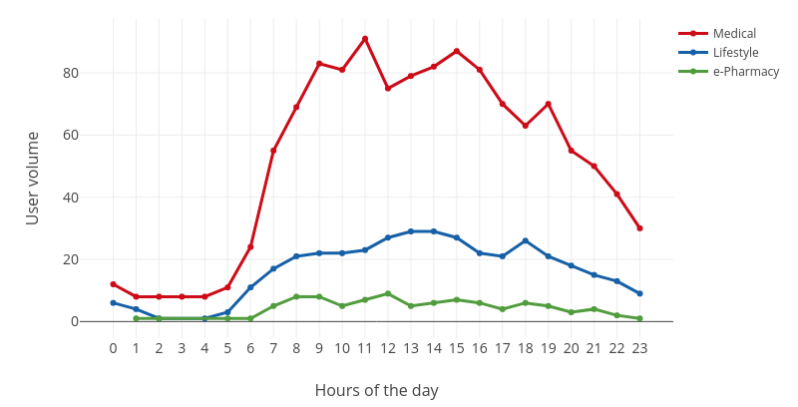
The fluctuation of the user volume in a day.

**Figure 7:**
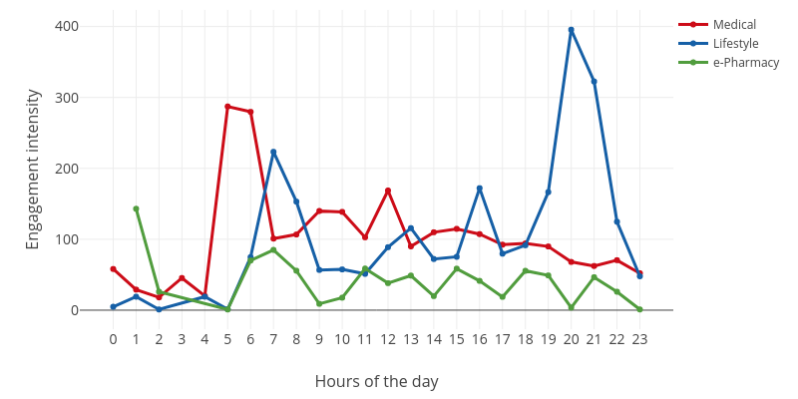
The fluctuation of the engagement intensity in a day.

The engagement trends at the day level are shown in Figures 5-7. As shown in Figures 5-7, user engagement increased gradually from morning to noon. After noon, user engagement declined until it reaches its lowest level at midnight. In other words, user engagement reached the highest level around noon and the lowest level at midnight. The main reason for this pattern is that users are prone to check their phones during midday lunch hours. In contrast, users engage the least at midnight because they fall asleep at that time.

For medical websites, user engagement peaked at noon. The highest engagement volume appeared at 12 noon, and the highest user volume appeared at 11 AM. However, peak engagement intensity occurred between 5 AM and 6 AM. One possible explanation is that users who encounter health problems at night will search for health information on the web during this period.

For lifestyle websites, the highest engagement was in the evening. For example, peak engagement volume and intensity occurred at 8 PM. This is because the use of lifestyle websites (eg, yoga exercise) usually takes a long time, and the ideal time is right after work. However, the largest number of users were engaged in lifestyle websites at 6 PM. Other peaks in engagement volume and intensity occurred at 7 AM, 1 PM, and 4 PM.

For e-pharmacy websites, user engagement fluctuated throughout the day. One special case is that the engagement intensity reaches its peak at 1 AM. e-Pharmacies are the most intensively used eHealth sites, and they have an engagement intensity that is even higher than those of the remaining two categories (ie, medical and lifestyle). One possible explanation for this phenomenon is that offline drug stores are closed at this time, and e-pharmacies are the only choice.

#### Customer Loyalty

Customer loyalty is a measure of a customer’s likelihood of engaging in repeat business with a company or brand. In this study, customer loyalty was measured using the following variables:

1. Total visits: the total number of visits within the observation period.
2. Visit days: the number of days visited within the observation period.
3. Average daily visits: the average number of visits per day.
4. Recency: the number of days since the last visit (in this study, recency was measured based on the difference between the last visit date and the end of the observation period).

**Figure 8:**
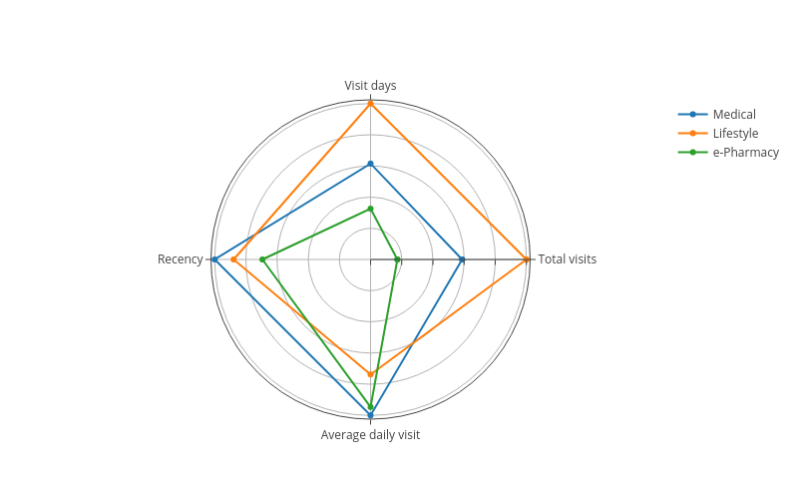
Customer loyalty.

A radar chart was used to present customers’ loyalty to the three categories of eHealth websites (Figure 8). A radar chart consists of a sequence of equiangular spokes called *radii*, with each spoke representing one of the variables. The data length of a spoke is proportional to the magnitude of the variable for the data point relative to the maximum magnitude of the variable across all data points. A line is drawn connecting the data values for each spoke. This gives the plot a starlike appearance and is the origin of one of the popular names for this plot.

The results in Figure 8 suggest that lifestyle websites, followed by medical websites, have the highest customer loyalty. e-Pharmacy websites have the lowest customer loyalty. This is because the service provided by lifestyle websites requires consistent user engagement over time (eg, fitness and chronic disease self-management). Medical websites also have relatively high customer loyalty because they act as portals to many health services such as health education, web-based consultation, and web-based registration. e-Pharmacies have the lowest customer loyalty because the demand for web-based drug purchases can often be fulfilled by offline pharmacies or hospitals. Many patients visit a web-based pharmacy only when they cannot obtain the drugs offline. In addition, different e-pharmacies are also substitutes for each other because the drugs they sell are standard products.

#### Cross-site Engagement

A user may visit several eHealth websites simultaneously, a phenomenon known as cross-site visits [22]. In this study, we were interested in two levels of cross-site engagement: user level and visit level.

At the user level, suppose that the number of users who visit lifestyle websites is x and the number of users who also visit medical websites is y. The cross-site engagement of lifestyle websites with medical websites is y/x. Cross-site engagement at the user level is shown in Table 4. The cross-site engagement of users of lifestyle websites to medical websites was 0.66, indicating that 66% (31/47, 66%) of lifestyle users also visited medical websites. Similarly, the cross-site engagement of users of e-pharmacy websites to medical websites was 0.92, and the cross-site engagement of users of e-pharmacy websites to lifestyle websites was 0.46. It should be noted that cross-site browsing was not symmetrical. As shown in Table 4, 66% (31/47, 66%) of lifestyle website users visited medical websites, whereas only 25% (31/124, 25%) of medical website users visited lifestyle websites. The asymmetric nature indicates that medical websites are more popular than lifestyle websites, and lifestyle websites are more popular than e-pharmacy websites.

**Table 4:** Cross-site engagement on the user level.

Category	Medical, n/N (%)	Lifestyle, n/N (%)	e-Pharmacy, n/N (%)
Medical	124/124 (100)	31/124 (25)	21/124 (17)
Lifestyle	31/47 (66)	47/47 (100)	11/47 (23)
e-Pharmacy	21/23 (92)	11/23 (48)	23/23 (100)

At the visit level, suppose that the number of users who visit the lifestyle websites is w, and the corresponding number of visits is u. On the same day, the number of visits to medical websites by these users is v, and the cross-site engagement of lifestyle websites to medical websites is v/u. Cross-site engagement at the visit level is shown in Table 5. The cross-site engagement of visits to lifestyle websites to medical websites was 0.94, indicating that 94% (44,223/46,870, 94%) of the visits to lifestyle websites were associated with visits to medical websites on the same day. Similarly, the cross-site engagement of visits to e-pharmacy websites to medical websites was 0.98, and the cross-site engagement of visits to e-pharmacy websites to lifestyle websites was 0.71. However, the cross-site engagement of visits was much lower for the opposite case. For example, the cross-site engagement of visits to medical websites to lifestyle websites was 0.33, the cross-site engagement of visits to medical websites to e-pharmacy websites was 0.03, and the cross-site engagement of visits to lifestyle websites to e-pharmacy websites was 0.06. This finding indicates that popular eHealth websites such as medical websites can effectively provide referral traffic for lifestyle and e-pharmacy websites. However, the opposite is not true. Lifestyle and e-pharmacy websites can provide only limited referral traffic for medical websites.

**Table 5:** Cross-site engagement on the visit level (n=373).

Category	Medical, n/N (%)	Lifestyle, n/N (%)	e-Pharmacy, n/N (%)
Medical	134,009/134,009 (100)	44,223/134,009 (33)	3868/134,009 (3)
Lifestyle	44,223/46,870 (94)	46,870/46,870 (100)	2812/46,870 (6)
e-Pharmacy	3868/3947 (98)	2812/3947 (71)	3947/3947 (100)

### Factors Influencing User Engagement

#### Overview

In this section, we investigate how the platform, channel, sex, and income influence user engagement (ie, engagement volume and engagement intensity) on eHealth websites. More specifically, we first investigate their influence independently and then investigate their interaction effects. Engagement volume is measured by the number of visits, and engagement intensity is measured by the number of visits per session (in this study, a session is defined as 1 day).

#### Platform

The platform refers to the operating system of the mobile phone used to visit eHealth websites. In this study, we focus on two platforms, iOS and Android, because they possess 97% of the global mobile market share. There are many differences between iOS and Android that may lead to different engagement behaviors on eHealth websites. Android has the greatest global market share at approximately two-thirds and has more app downloads than iOS. Sensor Tower reports that the Google Play Store experienced approximately 75.7 billion first-time app installs worldwide in 2018 [23]. Comparatively, the App Store experienced only 29.6 billion such installs. The Android platform also has more apps than the iOS platform (2.7 million Android apps vs 1.8 million iOS apps) [18]. In addition, Google Play Store has a higher percentage of free apps than the App Store [18].

iOS and Android also have different user groups. Owing to its broad price range and lower entry-level price point, Android has the largest global share in lower-income areas and developing nations [24]. It holds an advantage over iOS in emerging markets such as Asia and Africa. There is also a large gap between the purchasing power of an average iPhone user and that of an Android user. The median iPhone app user earns US $85,000 per year, which is 40% more than the median annual income of Android phone users (US $61,000) [24]. In addition, even though Android users have far more downloads than iOS users, iPhone users spend twice as much as their Android counterparts [24]. Android users also differ from iPhone users in their personalities. According to a recent study, Android users are less extroverted than iPhone users and are perceived to have greater levels of honesty and humility [25].

The results of the two-tailed *t* test comparing engagement volume and engagement intensity between Android and iOS users are shown in Table 6. In addition to the results of the *t* tests, we also report the effective sizes (small, medium, large, very large, and huge) to indicate the magnitudes of the differences. The effect size was first measured using Cohen *d* [26] and then interpreted as small (<0.01), medium (0.01-0.20), large (0.20-0.50), very large (0.50-0.80), or huge (0.80-2.0) according to the values of Cohen *d* [27]. The results in Table 6 show that Android users have a larger engagement volume (Cohen *d*=0.23; t_371_=2.26; *P*=.02, large effect size) and engagement intensity (Cohen *d*=1.39; t_371_=32.10; *P*<.001, large effect size) than iOS users.

**Table 6:** Comparison of user engagement between platforms^a^.

Platform engagement			Value, mean (SD)		*t* test (*df*)		*P* value		Cohen *d*
**Engagement volume**					2.26 (371)		.02		0.23
	Android	3.98 (2.08)							
	iOS	3.48 (2.22)							
**Engagement intensity**					32.10 (371)		<.001		1.39
	Android	3.29 (1.90)							
	iOS	1.18 (1.00)							

^a^Box-Cox transformation was applied to engagement volume and engagement intensity.

One possible explanation for the difference is that there are more health apps and fewer charges on Android. This makes it easier for Android users to find free health apps to satisfy their needs. In addition, Android users are more introverted and more proficient in information technology [25]. As a result, they are more willing to solve health concerns by visiting eHealth websites. In contrast, iPhone users may be more willing to go offline because of their health concerns.

#### Channel

The channel refers to the method through which a mobile user interacts with an eHealth website. In this study, we focused on two types of channels: mobile browsers and mobile apps. A browser can be found on any mobile phone, regardless of the operating system. Accessing an eHealth website through a browser is convenient because users do not need to download or install an app before the visit. However, it is essential to remember that network access, quality, and speed are all factors that can affect mobile web experience. Compared with a browser, an app has several advantages. For example, mobile apps offer greater personalization and operational efficiency, along with multiple other exclusive features. A well-designed mobile app can perform actions much quicker than a mobile website. In contrast to websites that generally use web servers, apps usually store their data locally on mobile devices. For this reason, data retrieval is quicker on mobile apps. Apps can further save users’ time by storing their preferences and using them to take proactive actions on their behalf. In addition, mobile apps can access and use built-in device features such as cameras, GPS, and location. Leveraging device capabilities leads to an enhanced, more convenient user experience.

We performed a *t* test to compare the engagement volume and engagement intensity between mobile browser users and mobile app users, and the results are shown in Table 7. The results in Table 7 show that app users have a larger engagement volume (Cohen *d*=1.44; t_371_=15.51; *P*<.001, large effect size) and engagement intensity (Cohen *d*=1.09; t_371_=21.51; *P*<.001, huge effect size) than browser users. The engagement volume of app users is 4.85 times that of browser users, and the engagement intensity of app users is 4.22 times that of browser users. Convenient access without remembering website URLs, faster and more fluent user experience, more powerful functions (eg, location-based service, alerts, and Quick Response code scanning), and more customization and personalization are all advantages of apps that may explain such a huge engagement gap.

**Table 7:** Comparison of user engagement between channels^a^.

User engagement and channel		Value, mean (SD)	*t* test (*df*)	*P* value	Cohen *d*
**Engagement volume**			15.51 (371)	<.001	1.44
	App	3.78 (2.13)			
	Browser	0.78 (0.76)			
**Engagement intensity**			21.51 (371)	<.001	1.09
	App	2.49 (1.76)			
	Browser	0.59 (0.57)			

^a^Box-Cox transformation was applied to engagement volume and engagement intensity.

#### Sex

The literature suggests that male and female users exhibit sizable differences in web-based engagement behaviors [17]. Therefore, sex may have some influence on eHealth website engagement. We performed a *t* test to compare the engagement volume and engagement intensity between female and male users, and the results are shown in Table 8.

**Table 8:** Comparison of user engagement between sexes^a^.

User engagement and sexes			Value, mean (SD)		*t* test (*df*)		*P* value		Cohen *d*
**Engagement volume**					0.82 (371)		.41		0.10
	Female	4.06 (2.46)							
	Male	3.83 (2.15)							
**Engagement intensity**					−8.36 (371)		<.001		0.38
	Female	2.14 (1.58)							
	Male	2.84 (1.93)							

^a^Box-Cox transformation was applied to engagement volume and engagement intensity.

The results in Table 8 suggest that the difference in the engagement volume between female and male users is not significant (Cohen *d*=0.10; t_371_=0.82; *P*=.41, medium effect size). However, male users had a greater engagement intensity (Cohen *d*=0.38; t_371_=−8.36; *P*<.001, large effect size) than female users. One possible explanation is that male users are more proficient in information technology skills (eg, internet information retrieval skills) [28].

#### Income

The literature also suggests that high-income and low-income users exhibit some differences in engagement behavior [17]. Therefore, income is also identified as a potential variable that may influence eHealth website engagement. In this study, income is measured using a proxy variable, monthly telecom expenditures. This is because data on actual income are difficult to obtain, and users with high incomes usually correspond to users with high telecom expenditures. Therefore, we performed a *t* test to compare the engagement volume and engagement intensity between low- and high-income users, and the results are shown in Table 9.

**Table 9:** Comparison of user engagement between low- and high-income users^a^.

User engagement and types of users			Value, mean (SD)		*t* test (*df*)		*P* value		Cohen *d*
**Engagement volume**					0.94 (371)		.35		0.11
	Low-income users	4.03 (2.34)							
	High-income users	3.79 (2.17)							
**Engagement intensity**					−1.07 (371)		.29		0.04
	Low-income users	2.57 (1.78)							
	High-income users	2.65 (1.85)							

^a^Box-Cox transformation was applied to engagement volume and engagement intensity.

The results in Table 9 suggest that the difference in the engagement volume between low- and high-income users is not significant (t_371_=0.94; *P*=.35). Furthermore, the difference in engagement intensity between low- and high-income users was not significant (t_371_=−1.07; *P*=.29). Therefore, we found no significant influence of income on the engagement patterns of eHealth websites.

#### Interaction Analysis

Interactions may exist among the four factors identified earlier. Therefore, an analysis of variance was conducted to test the potential interaction effects, and the results are shown in Table 10. The results in Table 10 indicate that the interaction between the platform and income is significant for both engagement volume (*F*_1,310_=6.20; *P*=.01) and engagement intensity (*F*_1,1817_=30.15; *P*<.001). This finding implies that although the main effect of income on engagement is not significant, it moderates the influence of the platform on engagement.

**Table 10:** The analysis of variance results^a^.

Factor		*F* test (*df*)	*P* value
**Engagement volume**			
	*Platform×income* ^b^	*6.20 (1)*	*.01*
	Channel×income	0.03 (1)	.86
	Platform×sex	1.36 (1)	.24
	Channel×sex	0.09 (1)	.76
**Engagement intensity**			
	*Platform×income*	*30.15 (1)*	*<.001*
	Channel×income	0.28 (1)	.60
	Platform×sex	0.12 (1)	.73
	Channel×sex	0.18 (1)	.67

^a^Box-Cox transformation was applied to engagement volume and engagement intensity.

^b^Italicization denotes significance (*P*<.05).

The details of the interaction between the platform and income are shown in Table 11. The results in Table 11 suggest that income negatively moderates the influence of the platform on engagement volume and engagement intensity. That is, the advantage of Android users over iOS users regarding engagement volume and engagement intensity is more salient among low-income users. More specifically, Android users have a significantly higher engagement volume and engagement intensity than iOS users when they are low income. However, Android users only have a significantly higher engagement intensity than iOS users when they are high income. For high-income users, the engagement volumes for Android users and iOS users were not significantly different. Low-income Android users are the users who are the most engaged in eHealth websites. Surprisingly, low-income iOS users are those who are least engaged in eHealth websites.

**Table 11:** The interaction between the platform and income^a^.

Income and platform		Engagement volume	Engagement intensity
**Low**			
	Android	4.55	2.86
	iOS	3.22	1.36
**High**			
	Android	3.97	2.41
	iOS	3.98	1.63

^a^Box-Cox transformation was applied to engagement volume and engagement intensity.

## Discussion

### Principal Findings

Several major findings were obtained in this study. First, the market share analysis indicates that the medical category accounts for the largest market share of eHealth website visits (134,009/184,826, 72.51%), followed by the lifestyle category (46,870/184,826, 25.36%). The e-pharmacy category had the smallest market share, accounting for only 2.14% (3947/184,826) of the total visits.

Second, eHealth websites are characterized by very low visit penetration but relatively high user penetration. This means that although eHealth websites are associated with a low usage frequency, they are closely related to everyone and have great market potential.

Third, the distribution of engagement intensity follows a power law distribution. A large number of eHealth needs involve only a small number of visits, whereas a very small number of complex eHealth needs must be realized through a large number of visits.

Fourth, visits to eHealth websites were highly concentrated. Most users (238/373, 63.8%) visited only one eHealth website within 3 months. On average, each user visits 1.5 eHealth websites. Fewer than 40% of users visit multiple eHealth websites.

Fifth, there are day and hour trends in eHealth website engagement patterns. User engagement is generally high on weekdays but low on weekends. In addition, user engagement increases gradually from morning to noon. After noon, user engagement declines until it reaches its lowest level at midnight.

Sixth, customer loyalty also differed significantly among the categories. Lifestyle websites, followed by medical websites, had the highest customer loyalty. e-Pharmacy websites had the lowest customer loyalty.

Seventh, cross-site browsing among categories was not symmetrical. For example, 66% (31/47, 66%) of lifestyle website users visited medical websites, whereas only 25% (31/124, 25%) of medical website users visited lifestyle websites. The asymmetric nature indicates that popular eHealth websites, such as medical websites, can effectively provide referral traffic for lifestyle and e-pharmacy websites. However, the opposite is not true.

Eighth, Android users are more engaged than iOS users on eHealth websites. This is because users can find more health apps that cost less on the Android platform. Another possible explanation is that Android users are more introverted or more proficient in information technology.

Ninth, app users are much more engaged than browser users. The engagement volume of app users is 4.85 times that of browser users, and the engagement intensity of app users is 4.22 times that of browser users. Such a sizable engagement gap can be explained by the great advantage of apps over browsers.

Tenth, male users had greater engagement intensity than female users. The engagement gap between male and female users can be explained by the fact that male users are more proficient in information technology skills.

Finally, income negatively moderates the influence of the platform (Android vs iOS) on user engagement. The advantage of Android users over iOS users regarding engagement volume and engagement intensity is more salient among low-income users. Low-income Android users are the users most engaged on eHealth websites. Conversely, low-income iOS users are those who are least engaged on eHealth websites.

### Limitations

This study also has some limitations. First, the sample size used in this study was not very large. Only 373 users from Shanghai, China, were included in the data set. More users should be incorporated in future analyses. Second, the income variable used in this study was measured using a proxy. It is measured by the monthly telecom expenditure. Although monthly expenditures should be associated with user income, their relationship is not deterministic. Better approaches, such as surveys, can be used to measure user income in future studies.

### Conclusions

In this study, we provide an overview of user engagement behavior on eHealth websites based on cross-site clickstream data. More specifically, we conducted an analysis to determine the market shares of different categories of eHealth websites, penetration of eHealth behavior, engagement intensity, engagement variety, day and hour trends, customer loyalty, and cross-site engagement behavior. Furthermore, we investigated the factors that influence user engagement on eHealth websites. The results indicate that the platform (Android vs iOS), channel (browser vs app), and sex (female vs male) have significant influences on engagement behavior. In addition, income (high vs low) negatively moderates the influence of platforms on engagement behavior.

Future research may focus on how the configuration of eHealth website resources may influence user engagement. Each eHealth website may have some health care resources (eg, health information, e-consultation, provider rating, and web-based registration). According to the resource orchestration theory, the role of one resource is not independent. Instead, its effect depends on the presence of other resources. How the configuration of resources may influence user engagement is an important RQ for the managers of eHealth websites. A configurational approach (eg, fuzzy set qualitative comparative analysis) can be used in the future to investigate the best resource composition pattern for eHealth websites.

## Abbreviations

RQ: research question
